# Preschoolers Focus on Others’ Intentions When Forming Sociomoral Judgments

**DOI:** 10.3389/fpsyg.2018.01851

**Published:** 2018-10-02

**Authors:** Julia W. Van de Vondervoort, J. Kiley Hamlin

**Affiliations:** Centre for Infant Cognition, Department of Psychology, The University of British Columbia, Vancouver, BC, Canada

**Keywords:** preschoolers, moral judgments, sociomoral judgments, helping, hindering, intention, outcome

## Abstract

Many studies suggest that preschoolers initially privilege outcome over intention in their moral judgments. The present findings reveal that, in contrast, even younger preschoolers can privilege intentions when evaluating characters who successfully or unsuccessfully help or hinder a third party in achieving its goal. Following a live-action puppet show originally created for infant populations, children made a forced-choice social judgment (which puppet was liked) and two forced-choice moral judgments (which puppet was nicer, which puppet should be punished), and were asked to explain their punishment allocations. In two experiments (*N* = 195), 3- and 4-year-olds evaluated characters with distinct intentions to help or to hinder who were associated with either positive or negative outcomes. Both ages judged characters with more positive intentions as nicer, and allocated punishment to characters with more negative intentions; neither of these tendencies depended on the outcomes the characters were associated with. Three-year-olds’ responses were somewhat less consistent than were 4-year-olds’, in that 3-year-olds’ judgments were disrupted by ambiguous harmful intent. Notably, children’s social judgments were less consistent than their moral judgments. In a third and final experiment (*N* = 100), children evaluated characters with the same intention but who were associated with different outcomes. Children showed inconsistent responding across age and outcome valence, but only 4-year-olds evaluating two characters with positive intentions reliably responded based on outcome. When providing informative responses in all three studies, children most frequently explained their punishment allocations by appealing to the puppet’s (attempted) hindering action or failure to help. These findings raise questions as to what underlies different patterns of response across studies in the literature, and suggests that observing live interactions may facilitate young children’s intention-based moral judgments.

## Introduction

When considering whether an action is good or praiseworthy versus bad or blameworthy, adults are sensitive to both an agent’s mental states (their intentions, beliefs, desires) and the outcomes they bring about. While in some cases adults do condemn those that unintentionally cause harm (e.g., Gino et al., 2008; Cushman et al., 2009; Cushman and Greene, 2012), adults typically privilege intentions over outcomes when making moral judgments (e.g., Malle, 1999; Mikhail, 2007; Young et al., 2007; Cushman, 2008). The ability to incorporate mental state information into moral judgments, rather than focus strictly on the outcomes of morally relevant actions, has long been considered a hallmark of moral maturity (Piaget, 1932/1965; Kohlberg, 1969).

Beginning with the work of Jean Piaget, researchers have explored when this feature of the mature moral sense becomes operational, and have documented a developmental transition whereby children’s moral judgments initially focus on outcomes and only later shift to focusing on intentions. For example, Piaget found that younger children tended to judge a child who accidently broke 15 cups as naughtier than a disobedient child who broke one cup, and it was not until age 8 – 10 that children focused on others’ intentions by more positively evaluating the child who accidentally caused a large negative consequence (1932/1965).

Subsequent studies revealed that Piaget’s methodology led him to underestimate the age at which children can use mental states to inform their moral judgments, suggestive that the centrality of intentions in moral judgments does not require many years of maturation, teaching, and/or relevant experiences to emerge. For example, young children incorporate intentions into their moral judgments when intentions are explicitly stated or otherwise made salient, when intentions are deconfounded from outcomes (e.g., consequences are held constant while intentions vary), and when a larger variety of test questions are used (e.g., asking about the agent rather than the acceptability of the act; e.g., Armsby, 1971; Buchanan and Thompson, 1973; Chandler et al., 1973; Costanzo et al., 1973; Farnill, 1974; Bearison and Isaacs, 1975; Berg-Cross, 1975; Karniol, 1978; Nelson-Le Gall, 1985; Cushman et al., 2013; Nobes et al., 2016). Under these circumstances, even 3-year-olds’ judgments show sensitivity to intentions (Nelson, 1980; Yuill, 1984; Nobes et al., 2009). That said, a host of studies have repeatedly demonstrated that young children initially privilege outcome over intention when the two are in conflict, and increasingly consider intention as they age (e.g., Armsby, 1971; Costanzo et al., 1973; Imamoglu, 1975; Moran and O’Brien, 1983; Zelazo et al., 1996; Helwig et al., 2001; Baird and Astington, 2004; Killen et al., 2011; Margoni and Surian, 2017; see also Li and Tomasello, 2018).

Notably, children’s ability to incorporate intentions into moral judgments has typically been tested using vignette-based tasks, in which experimenters narrate illustrated stories and then probe children’s explicit judgments (but see Chandler et al., 1973; Farnill, 1974, and Li and Tomasello, 2018 for use of videotaped scenes). These judgments include both verbal and Likert scale ratings of action acceptability (e.g., “Is it okay for [her] to [perform that act]? How good is it for [her] to [perform that act]? Is it really, really good, or just a little good, or just okay?”, Zelazo et al., 1996) and/or the moral worth of a character (e.g., “Is [he] a good boy or a bad boy?”, Costanzo et al., 1973).

Clearly, the tasks described above require that children can process a story presented verbally as well as respond to explicit questioning. These requirements may exclude or underrepresent the abilities of children who are unwilling or unable to engage in explicit questioning. Thus, researchers have recently developed tasks tapping more implicit forms of evaluation. Rather than asking for responses to specific test questions, some researchers have explored early sensitivity to others’ prosocial and antisocial intentions using age-appropriate behavioral tasks. Such studies provide further evidence that young children are sensitive to others’ intentions. For example, in one study 3-year-olds were less likely to help an adult who attempted but failed to harm a third-party compared to a neutral adult (Vaish et al., 2010), while in another study 3- and 4-year-olds were more likely to spontaneously correct punishments imposed on others who accidentally rather than intentionally caused the same harmful outcome (Chernyak and Sobel, 2016). In a final example, 5- and 6-year-olds used informants’ past intentions and outcomes when determining who to trust when searching for a prize (Liu et al., 2013).

Critically, more implicit forms of evaluation, in which neither the story presentations nor the response measures require verbal abilities, also allow for the study of preverbal children who are less than 3 years of age. To illustrate, in one study 5- and 9-month-old infants watched a live-action puppet show featuring a protagonist puppet who repeatedly tried but failed to open a box containing an attractive toy. In alternation, a helper puppet assisted the protagonist in opening the box so that he could access the toy, and a hinderer puppet slammed the box shut, preventing the protagonist from achieving his goal. When subsequently presented with a choice between the helper and hinderer, both 5- and 9-month-olds preferentially reached for the helper rather than the hinderer puppet, suggestive that infants differentially evaluated prosocial versus antisocial others (Hamlin and Wynn, 2011; for replications and related findings see Hamlin et al., 2007, 2010; Buon et al., 2014; Hamlin, 2015; Scola et al., 2015; Steckler et al., 2017; for failure to replicate see Salvadori et al., 2015; see also Scarf et al., 2012).

These implicit paradigms have also been utilized to explore infants’ sensitivity to third-party scenarios in which intentions and outcomes conflict. In one such task, 5- and 8-month-olds watched puppet shows in which successful and unsuccessful helpers and hinderers intervened following a protagonist’s repeated failure to open a box (Hamlin, 2013). The *successful* helper and hinderer achieved their respective goals to either assist or thwart the protagonist’s goal (as in Hamlin and Wynn, 2011). Conversely, the *failed* helper and hinderer brought about an outcome that conflicted with their intention: the failed helper tried but failed to open the box, while the failed hinderer tried but failed to prevent the protagonist from opening the box. When presented with different combinations of successful and failed helpers and hinderers, 8-month-olds preferentially reached for puppets with helpful intentions, regardless of the outcome that occurred (i.e., successful helpers over failed hinderers, failed helpers over successful hinderers, and failed helpers over failed hinderers). In contrast, when presented with two puppets who had demonstrated the same intention (i.e., successful helper and failed helper, failed hinderer and successful hinderer), 8-month-olds showed no preference for either puppet, suggestive that they did not evaluate characters based on the outcomes they were associated with. Unlike 8-month-olds, 5-month-olds preferentially reached for successful helpers over successful hinderers, but showed no preferences when presented with any failed puppet (Hamlin, 2013). Thus, infants’ sociomoral evaluations appear to privilege intentions over outcomes by 8 months of age, but not at 5 months (for related evidence with accidental help and harm see Hamlin et al., 2013; Woo et al., 2017).

In another task measuring infants’ expectations about characters involved in failed attempts to help and harm, 12- and 16-month-olds watched a video featuring a protagonist unsuccessfully attempting to climb a steep hill. Two characters alternately intervened: A successful hinderer who pushed the protagonist down the hill, and either a successful helper or unsuccessful helper (Lee et al., 2015). Subsequently, looking times suggested that 16-month-olds expected the protagonist to approach the character who had intended to help, even if he failed to do so and the protagonist’s outcome was negative. In contrast, 12-month-olds expected the protagonist to approach the successful helper rather than the hinderer, but only to approach the failed helper over the hinderer when outcome information was removed from the video (Lee et al., 2015). Together, these studies demonstrate that although a salient outcome may disrupt this sensitivity, infants are sensitive to others’ intentions to help or hinder – even when intentions and outcomes conflict. Indeed, unlike much work with young children (e.g., Armsby, 1971; Costanzo et al., 1973; Moran and O’Brien, 1983; Yuill, 1984; Zelazo et al., 1996; Helwig et al., 2001; Baird and Astington, 2004; Killen et al., 2011; Margoni and Surian, 2017), to our knowledge no infant studies to date have provided evidence that infants’ third-party social evaluations and expectations either rely solely on outcome or initially privilege outcome over intention.

What accounts for this apparent developmental discontinuity, whereby infants seem to privilege intentions but young children privilege outcomes? We reasoned that one possibility is that presentation of the social interactions via live puppet shows or videos, rather than via illustrated vignettes, might facilitate understanding, in that a fully acted-out scenario provides richer and more complete information than does a narrated short vignette (see Chandler et al., 1973; Farnill, 1974 for evidence that children are sensitive to the intentions of characters in videotaped scenes from age 6). If so, then presenting preschoolers with live puppet shows may facilitate relatively more mature moral reasoning – that is, positive evaluations of those with positive intentions and negative evaluations of those with negative intentions, irrespective of the eventual outcomes characters bring about.

The current studies explore whether young preschoolers’ social and moral judgments privilege intentions, even when the agents’ intentions conflict with the outcome of their actions. Scenarios were enacted via a live puppet show and based on shows previously utilized to explore infants’ sociomoral evaluations of characters with varying intention who are associated with varied outcomes (Hamlin, 2013). Children viewed events in which a protagonist unsuccessfully attempted to open a box to reach a toy inside (as in Hamlin and Wynn, 2011). Two additional puppets intervened: *helpers* demonstrated a positive intention to assist the protagonist, while *hinderers* demonstrated a negative intention to prevent the protagonist from achieving his goal. The helper and hinderer puppets were either *successful* in bringing about their objective, or *failed* to assist or thwart the protagonist’s goal. Thus, across studies, the protagonist interacted with four puppets: (1) successful helpers who try and help the protagonist achieve his goal, resulting in a positive outcome for the protagonist, (2) successful hinderers who try and block the protagonist’s goal, resulting in a negative outcome for the protagonist, (3) failed helpers who unsuccessfully try to help the protagonist achieve his goal, resulting in a negative outcome for the protagonist, and (4) failed hinderers who unsuccessfully try to block the protagonist’s goal, resulting in a positive outcome for the protagonist.

Each child was presented with two distinct events (e.g., failed helping and successful hindering), and then were asked three test questions: (1) which of the two puppets they “like,” (2) which was “nicer,” and (3) which “should get in trouble.” After children identified who should get in trouble, they were asked to explain this judgment. While these forced-choice questions do not allow conclusions regarding whether children (for example) think either puppet is “nice” (rather than “nicer”), these questions have been used to examine 3- to 5-year-olds’ social and moral judgments following helping and hindering puppet shows in which intentions and outcomes were not in conflict (Van de Vondervoort and Hamlin, 2017), are consistent with the forced-choice nature of infants’ evaluations in past work, and are consistent with questions previously used to explore young children’s explicit moral judgments (e.g., Costanzo et al., 1973; Zelazo et al., 1996; Baird and Astington, 2004; Cushman et al., 2013). Following the first round of liking/niceness/punishment test questions, children answered comprehension questions regarding the puppets’ actions and the outcome of each event and then answered the same test questions again. Comprehension questions ensured that children attended to both the failed/successful helper/hinderer’s actions and the outcome for the protagonist.

Experiment 1 explored whether 3- and 4-year-olds utilize actors’ mental states to inform their social and moral judgments when outcomes are equivalent. Children observed a live puppet show featuring a protagonist who failed to achieve his goal to open a box. In the “positive outcome” condition, a successful helper and failed hinderer intervened, resulting in a positive outcome for the protagonist (i.e., the box was opened and the toy was reached). In the “negative outcome” condition, a failed helper and successful hinderer intervened, always resulting in a negative outcome for the protagonist (i.e., the box was closed and the toy was not reached). Experiment 2 then examined whether 3- and 4-year-olds’ judgments privilege actors’ mental states or the outcome of their actions when these intentions and outcomes conflict. Children observed live puppet show events featuring the same protagonist; a failed helper intervened to bring about a negative outcome for the protagonist, while a failed hinderer was associated with a positive outcome. Finally, Experiment 3 investigated whether 3- and 4-year-olds’ judgments were sensitive to outcomes when actors’ mental states were equivalent. In one condition a successful helper and a failed helper intervened in the protagonist’s struggle, while in the second condition a successful hinderer and a failed hinderer intervened; critically, both puppets in each condition had the same intention but brought about opposite outcomes.

Based on work showing that young children are sensitive to intentions when outcomes are equivalent across scenarios in vignette tasks [e.g., Cushman et al. (2013) found that children evaluated a character who attempted but failed to cause harm more negatively than a character who successfully brought about an intended positive outcome by age 4; see also Chernyak and Sobel, 2016] and that infants privilege agents’ intentions following similar scenarios (Hamlin, 2013), we predicted that 3- and 4-year-olds in Experiment 1 would report liking the character with the positive intention, judge the character with the positive intention as nicer, and allocate punishment to the character with the negative intention, even though the characters were not distinguishable based on the valence of their associated outcomes. Further, based on work showing that 3-year-olds can have difficulty producing interpretable responses to open-ended questions (e.g., Kenward and Dahl, 2011; Van de Vondervoort and Hamlin, 2017), we predicted that in this and all further experiments, 4-year-olds would provide more informative verbal justifications than 3-year-olds. We also predicted that 4-year-olds would be more likely than 3-year-olds to reference sociomoral considerations as the reason for their punishment allocations, including references to the characters’ successful or unsuccessful attempts to block the protagonist’s goal. We did not predict that child’s gender would influence responding, but did explore whether females and males responded similarly in this and all further experiments, as this is common in developmental work (e.g., Helwig et al., 2001; Nobes et al., 2009).

## Experiment 1

### Method

#### Participants

Children in all experiments were recruited through hospitals and preschools in Vancouver, British Columbia and tested in a university research center or the child’s preschool. This and all other experiments were approved by the University of British Columbia’s Behavioral Research Ethics Board. Twenty-four 3-year-olds (*M*_age_ = 3;6, range = 3;2–3;11, 13 girls) and 24 4-year-olds (*M*_age_ = 4;6, range = 4;0–4;11, 16 girls) participated in the positive outcome condition, while 26 3-year-olds (*M*_age_ = 3;6, range = 3;0–3;11, 15 girls) and 24 4-year-olds (*M*_age_ = 4;4, range = 4;0–4;10, 12 girls) participated in the negative outcome condition. Before data collection began we established a pre-set stopping rule of 24 children per age per condition; two extra 3-year-olds were run due to scheduling issues. An additional 26 3-year-olds were tested but replaced due to failure to complete an English language warm-up (2), procedure error (1), unwillingness to participate (1), and a color and/or side preference that resulted in pointing to the same puppet across all test questions in one or both rounds (22). An additional eight 4-year-olds were tested but replaced due to color/side preferences. The decision to remove children that displayed a color/side preference in one or both rounds of test questions was pre-set following a pilot study, as children who judged that the same puppet is “liked,” “nicer” *and* “should get in trouble” appeared unmotivated and/or that they did not understand the test questions. The **Supplementary Materials** provide key analyses including children with color/side preferences; results are essentially identical in all experiments and do not influence the interpretations reported here. While demographic information was not formally collected, most participants in all experiments came from middle-class families representative of the racial and ethnic demographics of Vancouver, British Columbia.

#### Procedure

##### Warm-up

Children were shown a picture of a playground and asked to find the swing and slide, and to name the color of a toy and their favorite outside activity. Before data collection began it was decided that children would be replaced in the sample if they were unable/unwilling to locate the swing or slide via pointing; verbal responses were not required.

##### Puppet show

**FIGURE 1:**
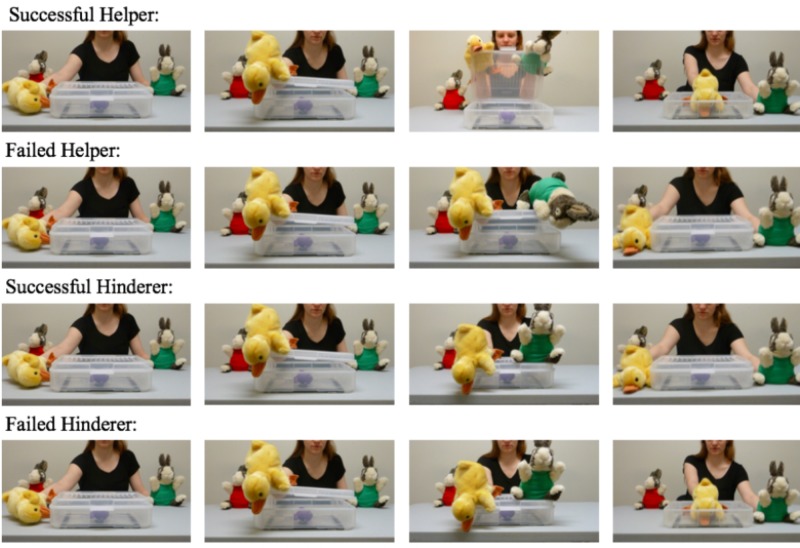
Visual depiction of the puppet show events. Written informed consent has been obtained from the depicted individual for the publication of these images.

Children participated in either the positive outcome condition or the negative outcome condition. All children watched a live puppet show featuring a protagonist struggling to achieve his goal to open a box and reach an attractive toy; a second and third puppet then intervened (successful helper and failed hinderer or failed helper and successful hinderer; see **Figure 1**). Puppet events were based on previous infant studies (Hamlin, 2013; see also Hamlin and Wynn, 2011), with two notable differences (as in Van de Vondervoort and Hamlin, 2017). First, for infants, the puppet events were enacted at the end of a long table and a curtain was lowered between events to hide the puppets; experimenters were hidden behind a curtain at the back of the table. Events in the current experiments were enacted on the floor or a table directly in front of the child and with the experimenter visible. Second, a few non-valenced words were added to the events for narration. All narrations were produced in a high-pitched, positive voice to indicate that the puppet was speaking rather than the experimenter; speech was not modulated based on the valence of the puppets’ intention or the eventual outcome.

Children watched four puppet events; two successful helper and two failed hinderer events in the positive outcome condition, two failed helper events and two successful hinderer events in negative outcome condition. At the start of each event, the successful/failed helper/hinderer puppets were seated on either side and back from a clear box containing a purple whale toy. The experimenter enacted the protagonist walking up to the box, looking through the side of the box while saying “Look, a toy!”, and unsuccessfully attempting to open the box five times. During the third to fourth attempt, the protagonist said, “Too heavy!”. On the fifth attempt, the successful/failed helper/hinderer intervened:

###### Successful helper

In successful helper events, the puppet ran forward, joined the protagonist’s struggle, and aided in opening the box while saying, “Open!” The puppet then ran away and the protagonist laid facedown, grasping the toy inside the box while saying “Toy!”

###### Failed helper

In failed helper events, the puppet ran forward and joined the protagonist’s attempts to open the box three more times; during the first attempt the puppet said “Open!” The puppet then ran away and the protagonist laid facedown beside the box while saying “No toy!”

###### Successful hinderer

In successful hinderer events, the puppet ran forward and jumped on the box, slamming it closed while saying “Close!” The puppet then ran away and the protagonist laid facedown beside the box while saying “No toy!”

###### Failed hinderer

In failed hinderer events, the puppet ran forward and jumped on the box, slamming it closed while saying “Close!” The protagonist then struggled to open the box while the puppet jumped on the box twice more^1^ before running away. After another struggle, the protagonist successfully opened the box and laid facedown, grasping the toy while saying “Toy!”

The narration during each event was designed to highlight the intervening puppets’ intention and the eventual outcome. Children were shown each event twice in a row, for a total of four events. Three puppets were used: a duck (protagonist) and two rabbits wearing a red and a green shirt (failed/successful helper/hinderer, identity counterbalanced). Additional counterbalanced variables were event order (red first, green first) and side of the puppets (red right, red left). For the question period puppets remained on the same side as during the show.

##### Test questions

Following the puppet events, children were presented with the successful/failed helper/hinderer puppets and asked (in counterbalanced order) which puppet they preferred (i.e., “Which one of these guys do you like the most?”) and which puppet was nicer (i.e., “Which one of these guys was nicer?”)^2^. To reduce response perseveration, children were asked to point to each puppet in between the liking and niceness questions (e.g., “Point to the guy with a red/green shirt. Right!”). Children were then asked which puppet deserved punishment and to explain this choice (i.e., “I think that one of these guys should get in trouble. Who should get in trouble? Why should he get in trouble?”). Children were prompted if they did not explain their punishment allocation (e.g., “What do you think?”). Children then answered comprehension questions and were asked the same test questions again. For each test question, children received a score of 1 if they responded in the direction of the hypothesis and 0 if not, resulting in a total of six scores (three test questions, two rounds of questioning) between 0 and 1 per child. One 4-year-old in the positive outcome condition responded that both puppets were liked in round one and one 3-year-old in the negative outcome condition responded, “I don’t know” when asked which puppet should be punished in round two; these responses were scored as against hypothesis.

##### Comprehension questions

Following the first round of test questions, children were shown each event type and asked one comprehension question about the intervening puppet’s action (e.g., for successful puppets, “Did he open the box or close the box?” and for failed puppets, “Did he try to open the box or try to close the box?”) and one comprehension question about the outcome for the protagonist (i.e., “Did the duck get the toy?”). If answered incorrectly, children were shown the event again and the comprehension question was repeated (e.g., “I don’t think he opened the box. Let me show you that one again”). If a comprehension question was answered incorrectly twice, children were corrected (e.g., “He opened the box. This bunny opened the box.”). Across experiments, 73% of children answered all four comprehension questions correctly the first time; only 3% of children required corrections before they answered test questions.

#### Transcription and Coding

When permitted by caregivers and possible within the preschool, participation was audio and visually recorded. A research assistant transcribed children’s verbal explanations from these recordings. When recording was not permitted (53 of 295 children across experiments), explanations were transcribed during the study by the experimenter. Two additional research assistants who were not involved in data collection or transcription coded children’s explanations according to the following categories:

##### Uninformative responses

Uninformative responses included those in which children provided no verbal response, unintelligible responses, or verbal responses that did not include a justification for the punishment allocation. These verbal responses included statements unrelated to the puppet events (e.g., “there’s a big storm”), statements without a justification (e.g., “because in trouble”), and statements that the child was unsure (e.g., “I don’t know”).

##### Informative responses

Informative responses were related to the shows and included:

###### Protagonist’s goal

References to the protagonist’s goal to open the box and/or reach the toy inside the box (e.g., “the duck [protagonist] was trying to open it”).

###### Relevant action

References to the puppet’s attempted or completed helping or hindering action (e.g., “he was trying to close the box,” “he closed the box,” “because she didn’t open it”).

###### Irrelevant action

References to positively or negatively valenced actions not from the shows (e.g., “because he punched this one”). While inaccurate, these responses may reflect children’s beliefs about actions that typically lead to punishment.

###### Relevant skill valence

References to the positive or negative nature of the puppet’s ability to open or close the box (e.g., “not strong,” “he wasn’t doing it very good”)^3^.

###### Relevant general valence

References to the positive or negative valence of the puppet or its actions that were related to the shows, but not related to the puppet’s ability to open or close the box (e.g., “he [selected puppet] was mean,” “this one [unselected puppet] was nicer”).

###### Irrelevant valence

References to the positive or negative valence of the puppet or its actions that were not directly related to the shows (e.g., “he’s mad”).

###### Non-social considerations

Responses that did not include sociomoral content, such as physical descriptions of the puppet (e.g., “he’s soft”), general disliking of the puppet (e.g., “I like the green shirt one”), descriptions of neutral acts (e.g., “he’s playing”), and ambiguous statements (e.g., “he does a lot of things”).

Each explanation was coded by two independent research assistants for the presence or absence of each response type; coders were blind to the referent (failed/successful helper/hinderer) of the explanation. Informative response types were not mutually exclusive. To avoid over-representing talkative children, whose explanations may have contained several types of informative responses, instances of each explanation type were represented as proportions and averaged across the two rounds. Reliability across the eight categories was strong (average Cohen’s kappa = 0.812; see McHugh, 2012). Disagreements were resolved by discussion among the two coders and the first author.

### Results

#### Test Questions

To explore whether responses differed before and after comprehension questions, we conducted a series of mixed-effect ANOVAs with round one scores and round two scores as within-subjects variables, and age (3, 4) and gender (female, male) as between-subjects factors. When compared to a Bonferroni-corrected alpha value of 0.017 (0.05/3), there were no main effects of round or interactions involving round of questioning within the positive outcome condition (all *F*s < 6.039, *p*s > 0.017, ${\text{η}}_{\text{p}}^{2}$s < 0.122) or the negative outcome condition (all *F*s < 3.437, *p*s > 0.069, ${\text{η}}_{\text{p}}^{2}$s < 0.069). Thus, children’s scores were summed across rounds resulting in three scores between 0 and 2 per child (liking, niceness, trouble scores). See the **Supplementary Materials** for scores in each round for all experiments; in all experiments, results from the first round are similar to those reported here and do not influence the interpretations presented in the main text. The dataset generated and analyzed for these experiments can be found on the Open Science Framework^4^.

##### Confirmatory analyses

**FIGURE 2:**
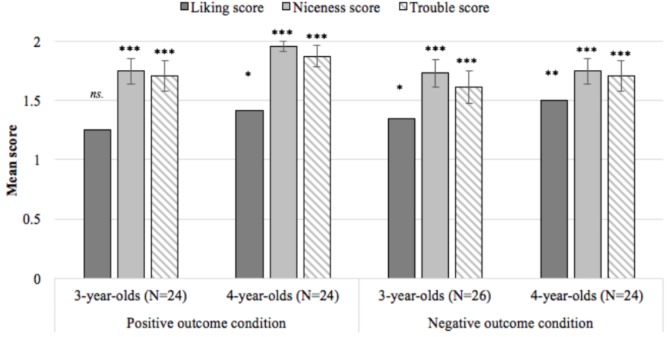
Mean liking, niceness, and trouble scores at each age in Experiment 1. Each score ranges between 0 and 2 with higher values indicating higher rates of with-hypothesis responding across two rounds of questioning; *^∗^p* < 0.05, *^∗∗^p* < 0.01, ^∗∗∗^*p* < 0.001, error bars reflect the standard error of the mean.

To determine at what age(s) liking, niceness, and trouble scores differed from chance, a series of one-sample *t*-tests compared scores at each age to a chance score of 1. Three-year-olds in the positive outcome condition did not distinguish between the puppets when reporting who they liked (*p* = 0.137), while 4-year-olds liked the successful helper (*p* = 0.015). Both ages judged the successful helper to be nicer (*p*s < 0.001) and allocated punishment to the failed hinderer (*p*s < 0.001). In the negative outcome condition, both ages liked the failed helper (*p*_3-year-olds_ = 0.047; *p*_4-year-olds_ = 0.005), judged the failed helper as nicer (*p*s < 0.001), and allocated punishment to the successful hinderer (*p*s < 0.001; see **Figure 2** and **Table 1** for descriptive and test statistics).

**Table 1:** Descriptive and test statistics for confirmatory analyses *t*-tests.

Experiment		1				2A		2B		3			
Condition		Positive outcome		Negative outcome						Positive intention		Negative intention	
Age		3	4	3	4	3	4	3	4	3	4	3	4
	*df*	23	23	25	23	23	23	23	24	23	25	22	26
Liking scores	*M*	1.250	1.417	1.346	1.500	1.167	1.542	1.125	1.320	1.000	1.462	0.913	0.741
	*SE*	0.162	0.158	0.166	0.159	0.177	0.159	0.174	0.170	0.181	0.149	0.165	0.137
	*t*	1.543	2.632	2.087	3.140	.941	3.406	0.720	1.877	0.000	3.094	0.526	1.892
	*d*	0.315	0.537	0.409	0.641	0.192	0.695	0.147	0.375	0.000	0.607	0.110	0.364
Nicer scores	*M*	1.750	1.958	1.731	1.750	1.208	1.792	1.583	1.880	1.375	1.423	0.913	1.037
	*SE*	0.109	0.042	0.118	0.109	0.159	0.120	0.119	0.088	0.157	0.138	0.153	0.155
	*t*	6.912	23.000	6.171	6.912	1.310	6.593	4.897	10.007	2.387	3.070	0.569	0.238
	*d*	1.411	4.695	1.210	1.411	0.267	1.346	1.000	2.001	0.487	0.602	0.119	0.046
Trouble scores	*M*	1.708	1.875	1.615	1.708	1.250	1.792	1.583	1.880	1.167	1.346	0.913	0.963
	*SE*	0.127	0.092	0.137	0.127	0.151	0.120	0.133	0.088	0.177	0.146	0.165	0.155
	*t*	5.560	9.559	4.500	5.560	0.827	6.593	4.371	10.007	0.941	2.368	0.526	0.238
	*d*	1.135	1.951	0.883	1.135	0.169	1.346	0.892	2.001	0.192	0.464	0.110	0.046

*Mean scores range between 0 and 2 with higher values indicating higher rates of with-hypothesis responding across two rounds of questioning*.

##### Exploratory analyses

To examine whether age, gender, and/or question type influenced children’s tendency to respond in the direction of the hypothesis, we conducted two mixed-effect ANOVAs with question type (liking, niceness, and trouble scores) as a within-subjects variable (repeated-measure), and age (3, 4) and gender (female, male) as between-subjects factors. In the positive outcome condition, there was only a main effect of question type (*F*[1.463,64.364] = 14.794, *p* < 0.001, ${\text{η}}_{\text{p}}^{2}$ = 0.252; all other *F*s < 1.542, *p*s > 0.220, ${\text{η}}_{\text{p}}^{2}$s < 0.035). To explore the main effect of question type, a series of paired-samples *t*-tests using the Bonferroni corrected alpha value of 0.017 (0.05/3) was used to compare scores on each question type across age. In the positive outcome condition, children were less likely to respond in the direction of the hypothesis when asked which puppet they liked (*M* = 1.333, *SE* = 0.113) compared to which puppet was nicer (*M* = 1.854, *SE* = 0.059; *t*[47] = 4.518, *p* < 0.001, *d* = 0.652) and which puppet should get in trouble (*M* = 1.792, *SE* = 0.079; *t*[47] = 4.276, *p* < 0.001, *d* = 0.617); there was no difference between niceness and trouble scores (*t*[47] = 1.000, *p* = 0.322, *d* = 0.144).

In the negative outcome condition, there was a main effect of question type (*F*[1.293,59.466] = 6.363, *p* = 0.009, ${\text{η}}_{\text{p}}^{2}$ = 0.122) and an interaction between age and gender (*F*[1,46] = 4.483, *p* = 0.040, ${\text{η}}_{\text{p}}^{2}$ = 0.089; all other *F*s < 0.846, *p*s > 0.362, ${\text{η}}_{\text{p}}^{2}$s < 0.019). To explore the main effect of question type, a series of paired-samples *t*-tests using the Bonferroni corrected alpha value of 0.017 (0.05/3) was used to compare scores on each question type across age. Children were again less likely to respond in the direction of the hypothesis when asked which puppet they liked (*M* = 1.420, *SE* = 0.115) compared to which puppet was nicer (*M* = 1.740, *SE* = 0.080; *t*[49] = 2.947, *p* = 0.005, *d* = 0.417) and which puppet should get in trouble (*M* = 1.660, *SE* = 0.093; *t*[49] = 2.585, *p* = 0.013, *d* = 0.366); again there was no difference between niceness and trouble scores (*t*[49] = 1.661, *p* = 0.103, *d* = 0.235). To explore the interaction between age and gender, two independent-samples *t*-tests using the Bonferroni corrected alpha value of 0.025 (0.05/2) were used to compare overall scores (summing liking, niceness, and trouble scores, resulting in a score between 0 and 6 for each child) across the three question types. Among 3-year-olds, males were more likely to respond in the direction of the hypothesis (*M* = 5.546, *SE* = 0.207) than were females (*M* = 4.067, *SE* = 0.530; *t*[18.024] = 2.600, *p* = 0.018, *d* = 0.945); there was no difference between 4-year-olds males’ (*M* = 4.667, *SE* = 0.620) and females’ scores (*M* = 5.250, *SE* = 0.392; *t*[22] = 0.796, *p* = 0.435, *d* = 0.339).

#### Punishment Explanations

Children at both ages most frequently appealed to relevant (un)successful helping or hindering actions when explaining their punishment allocations: 44% of 3-year-olds and 65% of 4-year-olds in the positive outcome condition, and 52% of 3-year-olds and 59% of 4-year-olds in the negative outcome condition did so (see **Table 2**). In their statements, nearly all children referenced the puppet’s attempted or completed hindering action (e.g., “he was closing it,” “he closed the lid”), although one 4-year-old in the negative outcome condition referenced a helping action (i.e., “he’s trying to open that” in round one, and “because he was opening the box” in round two when explaining why the failed helper should be punished).

**Table 2:** Proportions of explanations containing each response type in Experiments 1 – 3.

Experiment	Age	Uninformative responses	Informative responses							Relevant responses
			Protagonist’s goal	Relevant action	Irrelevant action	Relevant general valence	Relevant skill valence	Irrelevant valence	Non-social	
1 (positive outcome condition)	3	0.313 (0.089)	0.031 (0.022)	0.438 (0.095)	0.021 (0.021)	0.135 (0.062)	0	0.063 (0.046)	0	0.604 (0.095)
	4	0.125 (0.062)	0.021 (0.014)	0.646 (0.091)	0	0.146 (0.070)	0	0	0.063 (0.046)	0.813 (0.073)
1 (negative outcome condition)	3	0.077 (0.046)	0	0.519 (0.094)	0.039 (0.038)	0.154 (0.072)	0.019 (0.019)	0	0.192 (0.074)	0.692 (0.088)
	4	0.188 (0.066)	0.021 (0.021)	0.590 (0.087)	0.042 (0.042)	0.038 (0.027)	0	0	0.122 (0.053)	0.649 (0.087)
2A (opposing	3	0.396 (0.090)	0	0.292 (0.083)	0.094 (0.049)	0.115 (0.058)	NA	0.021 (0.021)	0.083 (0.049)	0.401 (0.094)
outcomes/intentions)	4	0.188 (.089)	0.028 (0.022)	0.424 (0.093)	0.063 (0.046)	0.247 (0.083)	NA	0	0.052 (0.043)	0.698 (0.089)
2B (opposing	3	0.271 (0.074)	0	0.385 (0.085)	0	0.198 (0.075)	0	0.083 (0.058)	0.063 (0.034)	0.583 (0.089)
outcomes/intentions)	4	0.040 (0.040)	0.050 (0.025)	0.790 (0.069)	0.020 (0.020)	0.080 (0.045)	0	0	0.020 (0.020)	0.920 (0.047)
3 (positive intention condition)	3	0.479 (0.097)	0	0.156 (0.064)	0.083 (0.049)	0.104 (0.060)	0.031 (0.023)	0.021 (0.021)	0.125 (0.062)	0.292 (0.085)
	4	0.404 (0.088)	0	0.423 (0.091)	0.019 (0.019)	0.019 (0.019)	0.058 (0.042)	0	0.077 (0.046)	0.500 (0.096)
3 (negative intention condition)	3	0.370 (0.095)	0.018 (0.013)	0.359 (0.089)	0.062 (0.046)	0.138 (0.066)	0	0	0.054 (0.031)	0.515 (0.097)
	4	0.259 (0.077)	0.019 (0.019)	0.343 (0.083)	0.056 (0.041)	0.167 (0.060)	0	0.019 (0.013)	0.139 (0.051)	0.528 (0.091)

*Proportion (SE). The informative response categories were not mutually exclusive and proportions sum to 1. Relevant responses include appeals to the protagonist’s goal, relevant actions, relevant general valence, and relevant skill valence*.

To test whether younger children provide less interpretable explanations, two factorial ANOVAs examined the effect of age (3, 4) and gender (female, male) on the proportion of uninformative responses across rounds. While the proportion of uninformative responses was greater among 3-year-olds compared to 4-year-olds in the positive outcome condition (*F*[1,44] = 4.117, *p* = 0.049, ${\text{η}}_{\text{p}}^{2}$ = 0.086; all other *F*s < 2.694, *p*s > 0.107, ${\text{η}}_{\text{p}}^{2}$s < 0.059), there was no difference in the proportion of uninformative responses across age in the negative outcome condition (*F*[1,46] = 1.665, *p* = 0.203, ${\text{η}}_{\text{p}}^{2}$ = 0.035; all other *F*s < 2.055, *p*s > 0.158, ${\text{η}}_{\text{p}}^{2}$s < 0.044).

Finally, to test whether 4-year-olds would be more likely than 3-year-olds to reference relevant sociomoral content when explaining their punishment allocations, we combined appeals to the protagonist’s goal, relevant actions, relevant general valence, and relevant skill valence into a single “relevant responses” category. A factorial ANOVA examining the effect of age (3, 4) and gender (female, male) on the proportion of relevant responses revealed that both ages provided equally relevant responses in both the positive outcome condition (*F*[1,44] = 2.867, *p* = 0.097, ${\text{η}}_{\text{p}}^{2}$ = 0.061; all other *F*s < 0.187, *p*s > 0.667, ${\text{η}}_{\text{p}}^{2}$s < 0.005) and the negative outcome condition (*F*[1,46] = 0.180, *p* = 0.674, ${\text{η}}_{\text{p}}^{2}$ = 0.004; all other *F*s < 0.551, *p*s > 0.461, ${\text{η}}_{\text{p}}^{2}$s < 0.013).

Overall, Experiment 1 demonstrated that preschoolers distinguish between characters with opposing intentions when outcomes are uninformative. When presented with a successful helper and failed hinderer who both brought about a positive outcome, 3-year-olds showed no preference for either character while 4-year-olds’ preferred the successful helper. Both 3- and 4-year-olds judged the successful helper to be nicer and allocated punishment to the failed hinderer. When presented with a failed helper and successful hinderer who both brought about a negative outcome, both 3- and 4-year-olds preferred the failed helper, judged the failed helper as nicer, and allocated punishment to the successful hinderer. Across conditions, children at both ages were more likely to respond in the direction of the hypothesis with respect to the moral questions (niceness/punishment) than the social questions (liking), and most often referenced the character’s attempted or completed hindering action when explaining which character should get in trouble.

Results from Experiment 1 are consistent with past work in which young children demonstrate sensitivity to others’ intentions when intentions do not conflict with the outcomes brought about (Buchanan and Thompson, 1973; Costanzo et al., 1973; Farnill, 1974; Nelson, 1980). Experiment 2 sought to determine whether children still privilege intentions when they do conflict with outcomes. Children observed a puppet show featuring a protagonist who unsuccessfully attempted to open a box. A failed helper and failed hinderer intervened; both characters brought about outcomes that conflicted with their intention. Based on past work showing that older preschoolers can incorporate intention information into their vignette-based judgments [e.g., Cushman et al. (2013) found that children evaluate accidental harm more positively than attempted harm by age 5], younger preschoolers’ sensitivity to intentions following puppet show events in Experiment 1, and past work showing that infants privilege agents’ intentions following these puppet events (Hamlin, 2013), we predicted that both 3- and 4-year-olds would report liking the character with the positive intention, judge the character with the positive intention as nicer, and allocate punishment to the character with the negative intention.

## Experiment 2A

### Method

#### Participants

Twenty-four 3-year-olds (*M*_age_ = 3;5, range = 3;0–3;11, 13 girls) and 24 4-year-olds (*M*_age_ = 4;6, range = 4;0–4;11, 9 girls) were tested in a university research center or the child’s preschool. An additional 15 3-year-olds were replaced due to unwillingness to participate (1), caregiver interference (1), failure to accept correction during comprehension questions (2), and a color/side preferences (11). An additional three 4-year-olds were replaced due to unwillingness to participate (1) and color/side preferences (2).

#### Procedure

The warm-up task, test questions, comprehension questions (i.e., “Did he try to open the box or try to close the box? Did the duck get the toy?”), transcriptions and coding procedures were identical to Experiment 1.

##### Puppet show

Children watched a live puppet show featuring a protagonist struggling to open a box; a second and third puppet intervened (failed helper in two events, failed hinderer in two events). All details were identical to those in Experiment 1, except for the actions of the failed hinderer:

###### Failed hinderer

In failed hinderer events, the puppet ran forward and jumped on the box, slamming it closed while saying “Close!” The puppet then ran away. After another struggle, the protagonist successfully opened the box and laid facedown, grasping the toy while saying “Toy!” Note that unlike in the positive outcome condition of Experiment 1, the present failed hinderer puppet jumped on the box once rather than three times; this mirrors the failed hinderer events shown to infants in Hamlin (2013).

### Results

#### Test Questions

A series of mixed-effect ANOVAs explored whether responses differed before and after comprehension questions; this revealed no main effects of round or interactions involving round of questioning on liking, niceness, or trouble scores (all *F*s < 5.686, *p*s > 0.020, ${\text{η}}_{\text{p}}^{2}$s < 0.115). Children’s scores were summed across rounds resulting in three scores between 0 and 2 per child (liking, niceness, trouble).

##### Confirmatory analyses

**FIGURE 3:**
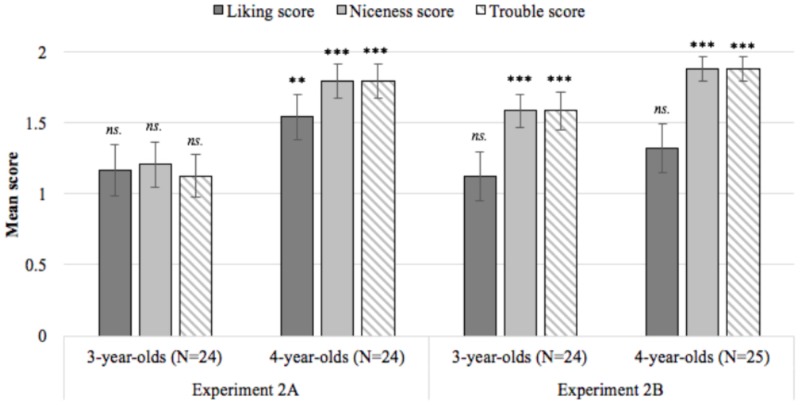
Mean liking, niceness, and trouble scores at each age in Experiments 2A and 2B. Each score ranges between 0 and 2 with higher values indicating higher rates of with-hypothesis responding across two rounds of questioning; ^∗^*p* < 0.05, ^∗∗^*p* < 0.01, ^∗∗∗^ *p* < 0.001, error bars reflect the standard error of the mean.

A series of one-sample *t*-tests comparing liking, niceness, and trouble scores at each age to a chance score of one revealed that younger children did not distinguish between the puppets: 3-year-olds’ liking (*p* = 0.357), niceness (*p* = 0.203), and trouble (*p* = 0.417) scores did not differ from chance. In contrast, 4-year-olds liked the failed helper (*p* = 0.002), judged the failed helper as nicer (*p* < 0.001), and allocated punishment to the failed hinderer (*p* < 0.001; see **Figure 3** and **Table 1**).

##### Exploratory analyses

A mixed-effect ANOVA was used to examine whether age, gender, and/or question type influenced children’s tendency to respond in the direction of the hypothesis. This revealed only a main effect of age (*F*[1,44] = 7.214, *p* = 0.010, ${\text{η}}_{\text{p}}^{2}$ = 0.141; all other *F*s < 2.449, *p*s > 0.114, ${\text{η}}_{\text{p}}^{2}$s < 0.054), such that 4-year-olds’ overall scores across the three questions types (*M* = 5.125, *SE* = 0.363) were higher than 3-year-olds’ (*M* = 3.500, *SE* = 0.421).

#### Punishment Explanations

The most frequent response among 3-year-olds were uninformative (40%), while their most informative responses were appeals to relevant (attempted) helping or hindering actions (29%); nearly all these appeals referenced a hindering action (e.g., “this one tried to close the box,” “because he closed it”), although one 3-year-old referenced a helping action in the second round (i.e., “because he’s trying to open” when explaining why the failed helper should be punished). The most frequent response among 4-year-olds were appeals to relevant (attempted) hindering actions (42%; see **Table 2**). A factorial ANOVA examined the effect of age and gender on the proportion of uninformative responses across rounds. While there was no main effect of age or gender (all *F*s < 2.396, *p*s > 0.128, ${\text{η}}_{\text{p}}^{2}$s < 0.053), there was an interaction between these factors (*F*[1,44] = 6.649, *p* = 0.013, ${\text{η}}_{\text{p}}^{2}$ = 0.131), such that 3-year-olds males (*M* = 0.546, *SE* = 0.142) provided more uninformative explanations than 4-year-old males [*M* = 0.067, *SE* = 0.067; *t*(14.377) = 3.047, *p* = 0.008, *d* = 1.373], while female 3-year-olds (*M* = 0.269, *SE* = 0.108) and 4-year-olds (*M* = 0.389, *SE* = 0.162) provided the same proportion of uninformative responses (*t*[20] = 0.642, *p* = 0.528, *d* = 0.292). Finally, a factorial ANOVA examining the effect of age and gender on the proportion of relevant sociomoral responses (protagonist’s goal, relevant actions, relevant general valence, and relevant skill valence) revealed that 4-year-olds provided more relevant responses than 3-year-olds (*F*[1,44] = 4.594, *p* = 0.038, ${\text{η}}_{\text{p}}^{2}$ = 0.095; all other *F*s < 1.695, *p*s > 0.199, ${\text{η}}_{\text{p}}^{2}$s < 0.038).

Overall, Experiment 2A reveals that 4-year-olds, but not 3-year-olds, privilege intentions when making social and moral judgments. When presented with a failed helper and failed hinderer, 4-year-olds preferred the failed helper, judged the failed helper to nicer, and allocated punishment to the failed hinderer. Four-year-olds’ explanations of their punishment allocations most frequently referenced the puppet’s (attempted) hindering action. In contrast, 3-year-olds failed to distinguish between the puppets when asked which puppet was liked, nicer, and should be punished. Given their chance responding to test questions, it is unsurprising that 3-year-olds’ explanations regarding punishment allocations were largely uninformative.

Given young children’s documented struggle to privilege intentions when intentions and outcomes conflict (e.g., Costanzo et al., 1973; Cushman et al., 2013), one possibility is that 3-year-olds simply do not use intentions to inform their sociomoral judgments when individuals can instead be distinguished by outcomes. Although 3-year-olds did not reliably distinguish characters by either intention or outcome, it is possible that they are in a transitional stage. An alternative possibility is that 3-year-olds can privilege intentions, but that the puppet shows in Experiment 2A did not adequately convey this mental state information to them. Specifically, the failed hinderer demonstrated his intention to close the box only once before the protagonist successfully opened it (in contrast, the failed helper demonstrated its intent three times); this may have made the strength of the failed hinderer’s negative intent somewhat ambiguous, rendering the distinction between the characters unclear.

Experiment 2B explored whether children privilege intentions when the failed hinderer’s intentions were made more salient. Children observed a failed helper and failed hinderer intervene in the protagonist’s struggle to open a box. As in Experiment 2A, the failed helper attempted to open the box three times. Unlike in Experiment 2A, the failed hinderer also demonstrated his negative intention three times, by repeatedly slamming the box closed. We predicted that both 3- and 4-year-olds would report liking the failed helper, judge the failed helper as nicer, and allocate punishment to the failed hinderer.

## Experiment 2B

### Method

#### Participants

Twenty-four 3-year-olds (*M*_age_ = 3;6, range = 3;0–3;11, 11 girls) and 25 4-year-olds (*M*_age_ = 4;6, range = 4;0–4;11, 12 girls) were tested in a university research center or the child’s preschool. The pre-set sample size was 24 children per age per condition; one extra 4-year-old was run due to scheduling issues. An additional 13 3-year-olds were replaced due to procedure errors (3) and color/side preferences (10). An additional five 4-year-olds were replaced due to procedure error (1) and color/side preferences (4).

#### Procedure

The warm-up task, test questions, comprehension questions (i.e., “Did he try to open the box or try to close the box? Did the duck get the toy?”), transcriptions and coding procedures were identical to previous experiments.

##### Puppet show task

Children watched a live puppet show featuring a protagonist struggling to open a box; a second and third puppet intervened (failed helper in two events, failed hinderer in two events). All puppet show details were identical to Experiment 1.

### Results

#### Test Questions

A series of mixed-effect ANOVAs explored whether responses differed before and after comprehension questions; this revealed no main effect of round on niceness or trouble scores, and no interactions involving round for liking, niceness, or trouble scores (Bonferroni-corrected alpha value of 0.017 [0.05/3]; all *F*s < 4.511, *p*s > 0.038, ${\text{η}}_{\text{p}}^{2}$s < 0.092). However, liking scores were higher after comprehension questions (*M* = 0.694, *SE* = 0.067) versus beforehand (*M* = 0.531, *SE* = 0.072; *F*[1,45] = 7.420, *p* = 0.009, ${\text{η}}_{\text{p}}^{2}$ = 0.142). Because round of questioning had no effect on liking scores in other experiments and consistently had no effect on niceness or trouble scores, children’s scores were summed across rounds resulting in three scores between 0 and 2 per child (liking, niceness, trouble).

##### Confirmatory analyses

A series of one-sample *t*-tests comparing liking, niceness, and trouble scores at each age to a chance score of one revealed that children did not prefer either the failed helper or hinderer: 3-year-olds’ liking scores (*p* = 0.479) and 4-year-olds’ liking scores (*p* = 0.073) did not differ from chance. However, both ages judged the failed helper to be nicer (*p*s < 0.001) and allocated punishment to the failed hinderer (*p*s < 0.001; see **Figure 3** and **Table 1**).

##### Exploratory analyses

A mixed-effect ANOVA examining whether age, gender, and/or question type influenced children’s tendency to respond in the direction of the hypothesis revealed a main effect of question type (*F*[1.208,54.356] = 14.550, *p* < 0.001, ${\text{η}}_{\text{p}}^{2}$ = 0.244; all other *F*s < 3.821, *p*s > 0.056, ${\text{η}}_{\text{p}}^{2}$s < 0.079). To explore this main effect, a series of paired-samples *t*-tests using the Bonferroni corrected alpha value of 0.017 (0.05/3) were used to compare scores on each question type across age. Children were less likely to respond in the direction of the hypothesis when asked which puppet they liked (*M* = 1.225, *SE* = 0.121) compared to which puppet was nicer (*M* = 1.735, *SE* = 0.076; *t*[48] = 3.900, *p* < 0.001, *d* = 0.557) and which puppet should get in trouble (*M* = 1.735, *SE* = 0.081; *t*[48] = 4.228, *p* < 0.001, *d* = 0.604); there was no difference between niceness and trouble scores (*t*[48] = 0.000, *p* = 1.000, *d* = 0.000).

#### Punishment Explanations

When asked to explain why the selected puppet should get in trouble, responses most frequently included appeals to relevant (attempted) helping or hindering actions: 39% of 3-year-olds and 79% of 4-year-olds (see **Table 2**). While these appeals typically referenced a hindering action (e.g., “because he tried to close the box”), one 4-year-old referenced the failed helper’s action in both rounds (i.e., “because he said open” when explaining why the failed helper should be punished). A factorial ANOVA examined the effect of age and gender on the proportion of uninformative responses across rounds and found that the proportion of uninformative responses was greater among 3-year-olds compared to 4-year-olds (*F*[1,45] = 7.438, *p* = 0.009, ${\text{η}}_{\text{p}}^{2}$ = 0.142; all other *F*s < 2.129, *p*s > 0.151, ${\text{η}}_{\text{p}}^{2}$s < 0.046). Finally, a factorial ANOVA examining the effect of age and gender on the proportion of relevant sociomoral responses (protagonist’s goal, relevant actions, relevant general valence, and relevant skill valence) revealed that 4-year-olds provided more relevant responses than 3-year-olds (*F*[1,45] = 11.502, *p* = 0.001, ${\text{η}}_{\text{p}}^{2}$ = 0.204; all other *F*s < 3.518, *p*s > 0.066, ${\text{η}}_{\text{p}}^{2}$s < 0.073).

Overall, Experiment 2B demonstrates that 3-year-olds can privilege intention over outcomes when making moral judgments when intentions are clarified (i.e., by having the failed hinderer demonstrate his intention to close the box three times rather than once). While 3-year-olds in Experiments 2A and 2B showed no preference for either the failed helper or failed hinderer, 3-year-olds in Experiment 2B judged the failed helper to be nicer and the failed hinderer to be more deserving of punishment. Four-year-olds in Experiment 2B also showed no preference for the failed helper or failed hinderer (c.f. Experiment 2A), but judged the failed hinderer as nicer and allocated punishment to the failed hinderer. This pattern suggests that, like in previous experiments, both 3- and 4-year-olds’ moral judgments (i.e., niceness, trouble) favor the failed helper more robustly than their social judgments (i.e., liking). The most frequent explanation for the allocation of punishment at both ages were references to the failed hinderer’s hindering action.

Experiment 3 explored whether children utilize outcomes to make social and moral judgments when characters’ intentions are the same. When intentions are completely uninformative, sociomoral judgments may favor individuals associated with positive versus negative outcomes, as these individuals may be associated with positive versus negative outcomes again in the future. Alternatively, judgments may favor individuals who are *successful* in bringing about their intended outcome, whatever it may be. Indeed, previous work has shown a relationship between judgments of competence and judgments of prosociality in children of this age (Stipek and Daniels, 1990; Brosseau-Liard and Birch, 2010; Landrum et al., 2016; but see Fusaro et al., 2011). That said, in a previous study infants tested with similar conditions did not distinguish characters who differed only on outcome (Hamlin, 2013).

In Experiment 3, children observed a puppet show featuring a protagonist unsuccessfully attempting to open a box. In the “positive intention” condition, a successful helper and failed helper intervened; both characters had a positive intention but the successful character brought about a positive outcome for the protagonist and the failed character brought about a negative outcome. In the “negative intention” condition, a successful hinderer and a failed hinderer intervened; both puppets had a negative intention but the successful character brought about a negative outcome for the protagonist and the failed character was associated with a positive outcome. Given previous work showing children’s bias toward outcomes when making sociomoral judgments, we predicted that both 3- and 4-year-olds would prefer characters who caused or were associated with positive outcomes. Thus, in the positive intention condition we predicted that children would prefer the successful helper, judge the successful helper to nicer, and allocate punishment to the failed helper, and in the negative intention condition we predicted children at both ages would prefer the failed hinderer, judge the failed hinderer as nicer, and allocate punishment to the successful hinderer.

## Experiment 3

### Method

#### Participants

Twenty-four 3-year-olds (*M*_age_ = 3;6, range = 3;0–3;10, 13 girls) and 26 4-year-olds (*M*_age_ = 4;5, range = 4;0–4;11, 14 girls) participated in the positive intention condition, while 23 3-year-olds (*M*_age_ = 3;6, range = 3;2–3;11, 13 girls) and 27 4-year-olds (*M*_age_ = 4;6, range = 4;0–4;11, 14 girls) participated in the negative intention condition. The pre-set sample size was 24 children per age per condition; five additional 4-year-olds were run due to scheduling issues and one child was initially recruited and tested as a 3-year-old but it was later learned that the child was 2-years-old at the time of testing. An additional 32 3-year-olds were tested but replaced due to unwillingness to participate (5), failure to complete an English language warm-up (1), and color/side preferences (26). An additional 17 4-year-olds were tested but replaced due to unwillingness to participate (2), failure to complete an English language warm-up (2), refusal to accept corrections following comprehension questions (1), and color/side preferences (12).

#### Procedure

The warm-up task, test questions, comprehension questions (for successful puppets, “Did he open the box or close the box? Did the duck get the toy?” and for unsuccessful puppets, “Did he try to open the box or try to close the box? Did the duck get the toy?”), transcriptions and coding procedures were identical to previous experiments. One 4-year-old in the positive intention condition indicated that neither puppet should get in trouble in round two, while one 3-year-old in the negative intention condition indicated neither puppet was liked or nicer in both rounds and three 4-year-olds indicated neither puppet was nicer in one or both rounds. These responses were scored as against the hypothesis that children would respond based on outcome.

##### Puppet show

Children watched a live puppet show featuring a protagonist struggling to open a box; a second and third puppet intervened (two successful helper events and two failed helper events in the positive intention condition, two successful hinderer events and two failed helper events in the negative intention condition). All puppet show details were identical to those in Experiment 1.

### Results

#### Test Questions

A series of mixed-effect ANOVAs explored whether responses differed before and after comprehension questions; this revealed no main effect of round or interactions involving round on liking, niceness, or trouble scores within the positive intention condition (Bonferroni-corrected alpha value of 0.017 [0.05/3]; all *F*s < 2.896, *p*s > 0.095, ${\text{η}}_{\text{p}}^{2}$s < 0.060) or the negative intention condition (all *F*s < 5.220, *p*s > 0.026, ${\text{η}}_{\text{p}}^{2}$s < 0.103). Children’s scores were again summed across rounds resulting in three scores between 0 and 2 per child (liking, niceness, trouble).

##### Confirmatory analyses

**FIGURE 4:**
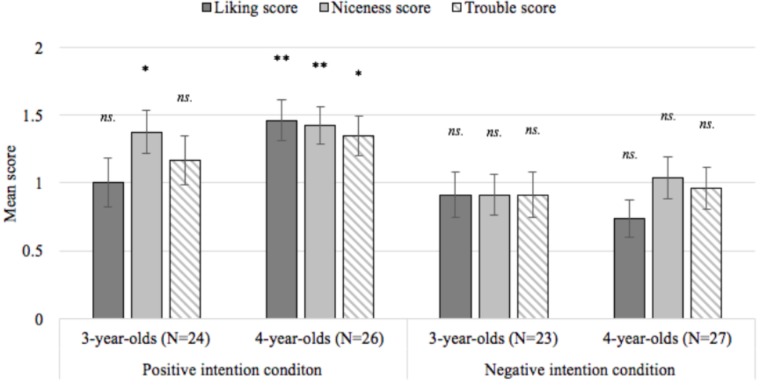
Mean liking, niceness, and trouble scores at each age in Experiment 3. Each score ranges between 0 and 2 with higher values indicating higher rates of with-hypothesis responding across two rounds of questioning; ^∗^*p* < 0.05, ^∗∗^*p* < 0.01, error bars reflect the standard error of the mean.

A series of one-sample *t*-tests comparing liking, niceness, and trouble scores at each age to a chance score of one revealed that 3-year-olds in the positive intention condition did not distinguish between the puppets when reporting who they liked (*p* = 1.000) or when allocating punishment (*p* = 0.357), though they did judge the successful helper to be nicer than the failed helper (*p* = 0.026). In contrast, 4-year-olds in the positive intention condition liked the successful helper (*p* = 0.005), judged the successful helper to be nicer (*p* = 0.005), and allocated punishment to the failed helper (*p* = 0.026). Children did not differentiate between the puppets for any test questions in the negative intention condition: 3- and 4-year-olds’ liking (*p*_3-year-olds_ = 0.604; *p*_4-year-olds_ = 0.070), niceness (*p*_3-year-olds_ = 0.575; *p*_4-year-olds_ = 0.814), and trouble scores (*p*_3-year-olds_ = 0.604; *p*_4-year-olds_ = 0.814, *d* = 0.046) did not differ from chance (see **Figure 4** and **Table 1**).

##### Exploratory analyses

Two mixed-effect ANOVAs revealed no effect of age, gender, or question type on children’s tendency to respond in the direction of the hypothesis in the positive or negative intention condition (all *F*s < 2.967, *p*s > 0.071, ${\text{η}}_{\text{p}}^{2}$s < 0.062).

#### Punishment Explanations

In the positive intention condition, 3-year-olds’ explanations regarding punishment allocation were mostly uninformative (48%). Four-year-olds also provided many uninformative responses (40%), and although neither puppet intended to close the box, 4-year-olds’ appeals to relevant actions most often referenced the puppet’s failure to help (e.g., “because she didn’t open it,” “he didn’t help the duck open the box”; 42%). In the negative intention condition, 3-year-olds’ responses were largely uninformative (37%) or appeals to relevant hindering actions (36%); 4-year-olds most often appealed to relevant hindering actions (34%), but also provided a number of uninformative responses (26%; see **Table 2**). Two factorial ANOVAs found no effect of age or gender on the proportion of uninformative responses across rounds in either condition (all *F*s < 1.359, *p*s > 0.249, ${\text{η}}_{\text{p}}^{2}$s < 0.030) and two factorial ANOVAs found no effect of age or gender on the proportion of relevant responses (protagonist’s goal, relevant actions, relevant general valence, and relevant skill valence) in either condition (all *F*s < 2.761, *p*s > 0.102, ${\text{η}}_{\text{p}}^{2}$s < 0.058).

Overall, Experiment 3 reveals that children’s social and moral judgments are not uniformly based on outcomes when intentions are identical. When positively intentioned characters brought about distinct outcomes, 3-year-olds judged the successful helper as nicer, but did not prefer or allocate punishment to either the failed or successful helper. Given their chance responding when asked which puppet should get in trouble, it is unsurprising that 3-year-olds’ explanations for this judgment were often uninformative. In contrast, 4-year-olds consistently utilized outcomes to inform their sociomoral judgments of positively intentioned characters (i.e., 4-year-olds liked the successful helper, judged the successful helper as nicer, and allocated punishment to the failed helper). Among 4-year-olds’ informative responses, explanations for punishment allocations most often referenced the puppet as having failed to help the protagonist, although neither puppet intended to thwart the protagonist’s goal. In contrast to the positive-intention condition, when negatively intentioned characters brought about distinct outcomes, both age groups responded at chance levels when asked which puppet was liked, nicer, and should get in trouble. When asked to explain their allocation of punishment, 3-year-olds’ responses were most frequently uninformative, while 4-year-olds most often appealed to the puppet’s attempted or completed hindering action.

## General Discussion

Experiments 1 – 3 provide evidence that 3- and 4-year-olds readily produce sociomoral judgments based on character’s intentions, rather than strictly on the outcomes these characters achieve. After observing live-action puppet shows in which characters’ intentions are fully acted out and the consequences of their actions can be directly observed, preschoolers were asked to provide a social judgment (i.e., which of two puppets is liked) and moral judgments (i.e., which of two puppets was nicer and which should be punished). Children were also asked to verbally justify their allocations of punishment. When characters could only be distinguished based on their intentions because outcomes were uninformative (Experiment 1), both 3- and 4-year-olds’ moral judgments revealed an intention focus, while children’s social judgments were less consistent: 4-year-olds, but not 3-year-olds, liked the successful helper over the failed hinderer, and both ages preferred the failed helper over the successful hinderer. When both intentions and outcomes conflicted in Experiment 2A, 4-year-olds’ social and moral judgments showed an intention focus, while 3-year-olds did not distinguish between the puppets. When the failed hinderer’s intention was further highlighted in Experiment 2B (i.e., the failed hinderer attempted to block the protagonist’s goal three times instead of once, the same number of attempts as the failed helper), children’s moral judgments showed a consistent focus on intention over outcome, though neither 3- nor 4-year-olds consistently preferred one character over the other. Finally, when characters had identical intentions but brought about opposing outcomes in Experiment 3, 4-year-olds’ social and moral judgments showed an outcome focus when comparing two characters with positive intentions, while 3-year-olds judged the successful helper to nicer than the failed helper and responded at chance when judging which character was preferred and which should receive punishment. Both ages responded at chance in all comparisons involving two negatively intentioned puppets.

Across all experiments, children’s most frequent informative justifications for their punishment allocation were appeals to the character’s hindering action. This was the case regardless of whether the action was successful or unsuccessful (e.g., children explained that a failed hinderer should get in trouble because he [tried to] block the protagonist’s goal), and whether the character had intended to bring about a negative outcome (e.g., when comparing a failed and successful helper, children explained that the failed helper should get in trouble because he did not allow the protagonist’s goal to be achieved). While we predicted that 3-year-olds’ would provide more uninformative responses than 4-year-olds (see Kenward and Dahl, 2011; Van de Vondervoort and Hamlin, 2017) and that 4-year-olds would be more likely than 3-year-olds to provide more relevant sociomoral considerations in their explanations, these predictions were largely unsupported.

These results provide evidence that young children can privilege intention over outcome when making moral judgments. Contrary to evidence suggesting that a focus on intentions develops after the early preschool years (Piaget, 1932/1965; see also Armsby, 1971; Costanzo et al., 1973; Moran and O’Brien, 1983; Yuill, 1984; Zelazo et al., 1996; Helwig et al., 2001; Baird and Astington, 2004; Killen et al., 2011; Margoni and Surian, 2017), both 3- and 4-year-olds’ forced-choice judgments regarding niceness and the allocation of punishment were based on which character displayed a positive versus negative intention to help or hinder a third party, regardless of the outcome achieved (except for 3-year-olds in Experiment 2A, in which the negatively intentioned character’s intention may have been unclear). This was the case when the characters being evaluated had opposing intentions but brought about the same outcome, and when both characters’ intentions and outcomes conflicted. Further, the consistency between children’s responding in the current study and infants’ responses to similar scenarios (Hamlin, 2013) suggests that sensitivity to others’ intentions develops earlier than previously thought.

Surprisingly, when outcomes were the only way to distinguish between characters, children did not consistently show an outcome bias: although 4-year-olds liked a successful helper over a failed helper, judged the successful helper to be nicer than the failed helper, and allocated punishment to the failed helper, they did not distinguish between a successful and failed hinderer on any test questions. Three-year-olds fared even worse, and responded above chance in the outcome conditions only when asked whether the successful versus failed helper was nicer. These results are surprising, both given past work suggestive of an outcome bias in this age group and because children could have alternatively distinguished between the characters based on which character successfully brought about their intended outcome (see Stipek and Daniels, 1990; Brosseau-Liard and Birch, 2010; Landrum et al., 2016 for evidence that young children’s judgments of competence and prosociality are related); however, (aside from some positive evaluation of the successful versus failed helper), children showed no consistent evidence of either strategy.

One potential concern is that 3-year-olds’ inability to distinguish between the failed and successful helpers when allocating punishment and both ages’ consistent failure to distinguish between the failed and successful hinderers may be due to the moral judgment questions asked. For instance, it is potentially unclear how to respond when asked which of two characters with positive intentions should get in trouble, or which of two characters with negative intentions is nicer. This ambiguity may have resulted in the observed chance-level responses in Experiment 3. However, it is important to note that these questions are only unclear if children are evaluating the puppets in light of their intentions. That is, if children were evaluating characters in terms of the outcomes they brought about, there would have been a clear answer to which of the two positively intentioned puppets was nicer (i.e., the successful helper) and to which of the two negatively intentioned puppets should get in trouble (i.e., the successful hinderer). Thus, it seems clear that children were not uniformly utilizing an outcome bias to answer these moral judgment questions, even when this was the only way that they might have distinguished between the characters.

Another potential concern is that the chance-level responding among 3-year-olds allocating punishment in the positive intention condition and among both 3- and 4-year-olds in the negative outcome condition of Experiment 3 is due to differences in how the same intentions were displayed across characters. Specifically, in the positive intention condition, the successful helper enacted his positive intention once (at which point he successfully aids the protagonist in opening the box) while the failed helper enacted his positive intention three times (i.e., by repeatedly struggling with the protagonist to try and open the box). Positive evaluations of both the successful helper’s positive outcome and the failed helpers’ repeated well-intentioned efforts may have resulted chance-level responding among 3-year-olds; 4-year-olds’ judgments favored the successful helper despite this concern. In the negative intention condition, the successful hinderer enacted his negative intention once before the protagonist’s goal is thwarted while the failed hinderer enacted his negative intention three times (i.e., by repeatedly slamming the box shut before the protagonist was eventually able to achieve his goal). Negative evaluations of both the outcome of the successful hinderer’s action and the failed hinderers’ repeated negative-intentioned efforts may have resulted in chance-level responding among 3- and 4-year-olds when comparing these two characters. It is possible that equating the intention displays (i.e., the successful helper tries to open the box three times before being successful, the successful hinderer slams the box closed three times before the protagonist fails to achieve his goal) would result in a consistent outcome bias when the characters’ intentions are equivalent. This possibility should be explored in future studies utilizing live-action puppet shows.

Regardless of children’s judgments when intentions are equivalent, the current studies show that 3- and 4-year-olds can use intentions to form moral judgments when outcomes are equivalent (Experiment 1) and can privilege intentions over outcomes when the two conflict (Experiment 2B). What accounts for children’s ability to privilege intentions following a live puppet show, compared to previous studies utilizing illustrated stories, in which young children initially fail to privilege intentions, especially when intentions and outcomes conflict (e.g., Killen et al., 2011; Cushman et al., 2013)? One possibility is that puppet shows allow characters’ intentions to be fully acted out, making intentions more salient than when explained during a vignette (even when intentions are explicitly stated). Likewise, processing demands may be reduced when children can observe the events unfold, rather than needing to infer what happened between images illustrating a vignette-based task. Finally, the pragmatic demands of puppet show-based tasks versus vignette-based tasks may account for differences in young children’s responding. For example, forced-choice comparisons between two puppets (e.g., asking which of two puppets is nicer) may allow children to distinguish between actors in a way that cannot be observed when children are instead asked to evaluate each character independently (e.g., asking whether each puppet is nice). Further, children’s pragmatic reasoning about the experimenter’s own intentions may lead them to focus on outcomes following vignettes if caregivers are more likely to use stories rather than pretend play to explain norms of behavior (e.g., stories depicting punishment for harms caused, regardless of the character’s intentions; see Westra and Carruthers, 2017 for a discussion of how children’s pragmatic reasoning may influence their performance on false-belief tasks). Future studies should probe these possibilities by directly comparing children’s judgments following vignette and puppet show versions of the same scenarios.

While the current studies provide evidence that children’s moral judgments are intention-based, 3- and 4-year-olds’ social judgments less consistently showed an intention-bias. Specifically, exploratory analyses revealed an effect of question type in the positive and negative outcome condition of Experiment 1 and in Experiment 2B, such that children were less likely to respond in the direction of the hypothesis (i.e., that children would favor the character with positive intentions over the character with negative intentions) when asked which character they liked, as opposed to when making moral judgments about niceness and punishment. These analyses suggest that the puppet show events were more consistently viewed as morally relevant as opposed to socially relevant, and that idiosyncratic preferences (e.g., preferences based on the puppet’s appearance) may have influenced children’s social judgments more than their moral judgments. Children in the current studies were only asked to explain one judgment to prevent contamination between explanations regarding the allocation of punishment and explanations regarding social preferences. That said, future studies should explore whether children’s social preferences are justified by appeals to the puppets’ helpful intention or by appeals to other aspects of the puppets or the events within the puppet show.

Finally, there are several remaining open questions regarding the developmental trajectory of sensitivity to others’ intentions. First, it is currently unknown whether infants’ implicit preferences for characters with positive intentions over characters with negative intentions, regardless of outcome (Hamlin, 2013), are related to preschoolers’ explicit sociomoral judgments following similar puppet show displays. While it is possible that infants’ implicit preferences and young children’s explicit judgments are distinct, it may be that sociomoral functioning in infancy is related to explicit moral development later in life. Relatedly, more work is needed to accurately characterize the use of intentions in moral judgments across the lifespan. This could be accomplished by utilizing the same stimuli to examine intention-based judgments in infants, preschoolers, older children, and adults. While the current studies adapted live puppet show stimuli previously shown to infants, practical concerns restricted our sample to the preschool years, rather than the broader age range necessary to make strong conclusions regarding the continuity of intention sensitivity across the lifespan. Lastly, it is also an open question whether an early focus on intentions is universal, and if so, how this develops into adult-like moral responses across a variety of cultures. Given variability in the extent to which adults from small-scale, non-Western societies incorporate intentionality in moral judgments (Barrett et al., 2016), it is possible that early moral judgments differ along important dimensions, or that infants and young children in both Western and non-Western share an early sensitivity to intentions that is refined according to their culture. Exploring the development of implicit evaluations and explicit judgments within and across diverse individuals over time would greatly contribute to our understanding of how intention and outcome information becomes integrated in mature moral judgments.

## Ethics Statement

These studies were developed and conducted in accordance with ethical guidelines and the protocols were approved by the University of British Columbia’s Behavioral Research Ethics Board. Caregivers gave written informed consent in accordance with the Declaration of Helsinki.

## Author Contributions

JV and JH developed the study hypothesis and design. Testing, data collection, and data analysis were performed by JV, who also drafted a first manuscript. JH provided critical revisions. JV and JH approved the final version of the manuscript for submission.

## Conflict of Interest Statement

The authors declare that the research was conducted in the absence of any commercial or financial relationships that could be construed as a potential conflict of interest.

## Footnotes

1 In the failed hinderer events in Hamlin (2013) the puppet jumped on the box once; here we equated the number of failed attempts (three) across the failed helper and failed hinderer events. See Experiment 2 in the current paper for children’s judgments of the failed hinderer following one versus three attempts to close the box.

2 After children identified the nicer puppet, they were asked whether that puppet was a “little bit nice or a lot nice” (order counterbalanced). We initially planned to examine niceness judgments on a 3-point scale from “not nice” to “a lot nice,” but because we did not train children on this scale prior to testing and because most children at each age responded that the selected puppet was “a lot” nice regardless of which puppet they indicated was nicer, this question is not considered further.

3 A need for this category had not been identified when the positive intention condition of Experiment 3 was initially coded. The category was added to the coding scheme once it was clear that some children were using skill explanations and the data was entirely recoded without discussing any statements within that condition. Due to the order in which the data was coded, this category was not included in the coding scheme for Experiment 2A; inspection of the transcriptions by the first author revealed it was not necessary to recode as no children used skill explanations. The full coding scheme including skill explanations was utilized for all other experiments and conditions.

4 https://osf.io/mgzq7/?view_only=903f3a74292940ee92312a2edb3aa7be
