# Supplementary material for: Preschoolers Focus on Others’ Intentions When Forming Sociomoral Judgments
Source: Front Psychol. 2018 Oct 2;9:1851. doi: 10.3389/fpsyg.2018.01851 (PMC6176058; doi:10.3389/fpsyg.2018.01851)
Supplement: Supplementary file 1 [file Table_1.docx]

**Supplementary Materials**

**1 Excluded Participants**

Following a pilot study, we decided that in all experiments we would remove and replace children that indicated the same puppet was “liked”, “nicer”, and “should get in trouble” in one or both rounds of test questions. Indicating the same puppet across positively and negatively valenced test questions suggested that children were unmotivated and/or did not understand the test questions. Further, since neither the color of the puppets’ shirts (red, green) nor their location (right, left) was varied across test questions within the same child, these children’s responses may have reflected a side and/or color preference. Confirmatory analyses including these children’s social and moral judgments are reported here. Overall, response patterns are very similar when children with a side/color preference are included in the analyses.

**2 Experiment 1**

**2.1 Method**

**2.1.1 Participants**

The full sample included 72 3-year-olds (*M*= 3;6, range = 3;0-3;11, 39 girls) and 56 4-year-olds (*M*= 4;5, range = 4;0-4;11, 32 girls).

**2.1.2 Procedure**

See the main text for procedural details for all experiments.

**2.2 Results**

To explore whether responses differed before and after comprehension questions, we conducted a series of mixed-effect ANOVAs with round one scores and round two scores as within-subjects variables, and age (3, 4) and gender (female, male) as between-subjects factors. When compared to a Bonferroni-corrected alpha value of .017 (.05/3), there was no main effects of round or interactions involving round of questioning on liking, niceness, or trouble scores in the negative outcome condition (all *F*s < 4.853, all *p*s > .030, all *η_p_^2^*s < .073). However, in the positive outcome condition, niceness scores were higher after comprehension questions (*M* = .918, *SE* = .035) versus beforehand (*M* = .787, *SE* = .053; *F*[1,57] = 6.636, *p* = .013, *η_p_^2^* = .104; all other *F*s < 1.446, all *p*s > .233, all *η_p_^2^*s < .026). Because round of questioning had no effect on niceness scores in other experiments and consistently had no effect on liking or trouble scores, children’s scores were summed across the two rounds resulting in three scores between 0 – 2 per child (liking, niceness, trouble).

A series of one-sample t-tests comparing children’s liking, niceness, and trouble scores at each age to a chance score of one revealed the same pattern of results as in the main text. Three-year-olds in the positive outcome condition did not distinguish between the puppets when reporting who they liked (*M* = 1.031, *SE* = .145; *t*[31] = .215, *p* = .831, *d* = .038), while 4-year-olds liked the successful helper (*M* = 1.379, *SE* = .144; *t*[28] = 2.635, *p* = .014, *d* = .489). Both ages judged the successful helper to be nicer (*M*_3-year-olds_ = 1.563, *SE* = .118; *t*[31] = 4.756, *p* < .001, *d* = .841; *M*_4-year-olds_ = 1.862, *SE* = .082; *t*[28] = 10.524, *p* < .001, *d* = 1.954) and allocated punishment to the failed hinderer (*M*_3-year-olds_ = 1.656, *SE* = .106; *t*[31] = 6.171, *p* < .001, *d* = 1.091; *M*_4-year-olds_ = 1.690, *SE* = .123; *t*[28] = 5.625, *p* < .001, *d* = 1.045). Both ages in the negative outcome condition liked the failed helper (*M*_3-year-olds_ = 1.375, *SE* = .132; *t*[39] = 2.831, *p* = .007, *d* = .448; *M*_4-year-olds_ = 1.519, *SE* = .145; *t*[26] = 3.578, *p* = .001, *d* = .689), judged the failed helper as nicer (*M*_3-year-olds_ = 1.675, *SE* = .097; *t*[39] = 6.936, *p* < .001, *d* = 1.097; *M*_4-year-olds_ = 1.667, *SE* = .119; *t*[26] = 5.586, *p* < .001, *d* = 1.075), and allocated punishment to the successful hinderer (*M*_3-year-olds_ = 1.300, *SE* = .130; *t*[39] = 2.306, *p* = .027, *d* = .365; *M*_4-year-olds_ = 1.630, *SE* = .121; *t*[26] = 5.199, *p* < .001, *d* = 1.001).

**3 Experiment 2A**

**3.1 Method**

**3.1.1 Participants**

The full sample included 35 3-year-olds (*M*= 3;5, range = 3;0-3;11, 16 girls) and 26 4-year-olds (*M*= 4;6, range = 4;0-4;11, 10 girls).

**3.2 Results**

A series of mixed-effect ANOVAs explored whether responses differed before and after comprehension questions; this revealed no main effects of round or interactions involving round of questioning on liking, niceness, or trouble scores (Bonferroni-corrected alpha value of .017 [.05/3]; all *F*s < 2.414, all *p*s > .125, all *η_p_^2^*s < .042). Children’s scores were summed across the two rounds resulting in three scores between 0 – 2 per child (liking, niceness, trouble).

A series of one-sample t-tests comparing children’s liking, niceness, and trouble scores at each age to a chance score of one revealed the same pattern of results as in the main text. Younger children did not distinguish between the puppets: 3-year-olds’ liking (*M* = 1.171, *SE* = .139; *t*[34] = 1.234, *p* = .226, *d* = .209), niceness (*M* = 1.171, *SE* = .126; *t*[34] = 1.358, *p* = .183, *d* = .230), and trouble (*M* = 1.086, *SE* = .132; *t*[34] = .649, *p* = .521, *d* = .110) scores did not differ from chance. In contrast, 4-year-olds liked the failed helper (*M* = 1.500, *SE* = .159; *t*[25] = 3.138, *p* = .004, *d* = .615), judged the failed helper as nicer (*M* = 1.731, *SE* = .131; *t*[25] = 5.588, *p* < .001, *d* = 1.096), and allocated punishment to the failed hinderer (*M* = 1.731, *SE* = .118; *t*[25] = 6.171, *p* < .001, *d* = 1.210).

**4 Experiment 2B**

**4.1 Method**

**4.1.1 Participants**

The full sample included 34 3-year-olds (*M*= 3;6, range = 3;0-3;11, 16 girls) and 29 4-year-olds (*M*= 4;5, range = 4;0-4;11, 13 girls).

**4.2 Results**

A series of mixed-effect ANOVAs explored whether responses differed before and after comprehension questions; this revealed no main effects of round or interactions involving round of questioning on liking, niceness, or trouble scores (Bonferroni-corrected alpha value of .017 [.05/3]; all *F*s < 5.441, all *p*s > .022, all *η_p_^2^*s < .085). Children’s scores were summed across the two rounds resulting in three scores between 0 – 2 per child (liking, niceness, trouble).

A series of one-sample t-tests comparing children’s liking, niceness, and trouble scores at each age to a chance score of one revealed a similar pattern of results as in the main text. As in the main text, 3-year-olds’s liking scores (*M* = 1.265, *SE* = .142) do not differ from chance (*t*[33] = 1.864, *p* = .071, *d* = .320). However, while in the main text 4-year-olds also showed no preference for either puppet, we now see that 4-year-olds prefer the failed helper (*M* = 1.310, *SE* = .150; *t*[28] = 2.073, *p* = .048, *d* = .385). As in the main text, both ages reliably judged the failed helper to be nicer (*M_3-year-olds_* = 1.529, *SE* = .105; *t*[33] = 5.022, *p* < .001, *d* = .861; *M_4-year-olds_* = 1.793, *SE* = .104; *t*[28] = 7.636, *p* < .001, *d* = 1.418) and allocated punishment to the failed hinderer (*M_3-year-olds_* = 1.382, *SE* = .127; *t*[33] = 3.016, *p* = .005, *d* = .517; *M_4-year-olds_* = 1.828, *SE* = .087; *t*[28] = 9.519, *p* < .001, *d* = 1.768).

**5 Experiment 3**

**5.1 Method**

**5.1.1 Participants**

The full sample included 73 3-year-olds (*M*= 3;6, range = 3;0-3;11, 36 girls) and 65 4-year-olds (*M*= 4;5, range = 4;0-4;11, 32 girls).

**5.2 Results**

A series of mixed-effect ANOVAs explored whether responses differed before and after comprehension questions; this revealed no main effects of round or interactions involving round of questioning on liking, niceness, or trouble scores in the positive (Bonferroni-corrected alpha value of .017 [.05/3]; all *F*s < 2.666, all *p*s > .106, all *η_p_^2^*s < .040) or negative intention condition (all *F*s < 2.900, all *p*s > .093, all *η_p_^2^*s < .044).

A series of one-sample t-tests comparing children’s liking, niceness, and trouble scores at each age to a chance score of one revealed a similar pattern of results to those reported in the main text. While in the positive intention condition of the main text, 3-year-olds distinguished between the successful helper and the failed helper for one test question and judged the successful helper to be nicer, they did not distinguish between the puppets for any test question in the full sample: 3-year-olds responded at chance when asked which puppet was liked (*M* = .919, *SE* = .136; *t*[36] = .595, *p* = .556, *d* = .098), nicer (*M* = 1.243, *SE* = .125; *t*[36] = 1.946, *p* = .059, *d* = .320), and should receive punishment (*M* = 1.108, *SE* = .133; *t*[36] = .813, *p* = .422, *d* = .134). As in the main text, 4-year-olds in the positive intention condition liked the successful helper (*M* = 1.424, *SE* = .138; *t*[32] = 3.078, *p* = .004, *d* = .536), judged the successful helper to be nicer (*M* = 1.394, *SE* = .130; *t*[32] = 3.028, *p* = .005, *d* = .527), and allocated punishment to the failed helper (*M* = 1.364, *SE* = .122; *t*[32] = 2.988, *p* = .005, *d* = .520).

As in the main text, children did not differentiate between the puppets for any test questions in the negative intention condition: 3- and 4-year-olds’ liking (*M*_3-year-olds_ = 1.083, *SE* = .140; *t*[35] = .595, *p* = .556, *d* = .099; *M*_4-year-olds_ = .781, *SE* = .133; *t*[31] = 1.648, *p* = .109, *d* = .291), niceness (*M*_3-year-olds_ = 1.083, *SE* = .128; *t*[35] = .649, *p* = .520, *d* = .108; *M*_4-year-olds_ = 1.000, *SE* = .142; *t*[31] = .000, *p* = 1.000, *d* = .000), and trouble scores (*M*_3-year-olds_ = .944, *SE* = .119; *t*[35] = .466, *p* = .644, *d* = .078; *M*_4-year-olds_ = 1.000, *SE* = .142; *t*[31] = .000, *p* = 1.000, *d* = .000) did not differ from chance.

**6 Results by Round (Main Text)**

The following table presents the number of children at each age in each condition answering in the direction of the hypothesis in round one (before answering comprehension questions) and in round two (after answering comprehension questions). These children did not display a color/side preference. To determine whether responses deviated from chance, a binomial p value was calculated for each question; significant values are noted below.

Supplementary Table 1.

*Number of with hypothesis responses before and after comprehension questions*

| Experiment | Condition | Age | N | Round 1 | | | Round 2 | | |
| --- | --- | --- | --- | --- | --- | --- | --- | --- | --- |
|  |  |  |  | Liking Question | Niceness Question | Trouble Question | Liking Question | Niceness Question | Trouble Question |
| 1 | Positive outcome | 3 | 24 | 15 | 19** | 19** | 15 | 23*** | 22*** |
|  |  | 4 | 24 | 16 | 23*** | 22*** | 18* | 24*** | 23*** |
|  | Negative outcome | 3 | 26 | 16 | 22** | 20* | 19* | 23*** | 22** |
|  |  | 4 | 24 | 17 | 20** | 19** | 19** | 22*** | 22*** |
| 2A |  | 3 | 24 | 14 | 14 | 14 | 14 | 15 | 13 |
|  |  | 4 | 24 | 18* | 22*** | 22*** | 19** | 21** | 21** |
| 2B |  | 3 | 24 | 10 | 19** | 18* | 17 | 19** | 20** |
|  |  | 4 | 25 | 16 | 23*** | 23*** | 17 | 24*** | 24*** |
| 3 | Positive intention | 3 | 24 | 12 | 16 | 13 | 12 | 17 | 15 |
|  |  | 4 | 26 | 20* | 19* | 19* | 18 | 18 | 16 |
|  | Negative intention | 3 | 23 | 10 | 12 | 13 | 11 | 9 | 8 |
|  |  | 4 | 27 | 11 | 14 | 13 | 9 | 14 | 13 |

Note. * < .05; ** <.01; *** < .001.
